# Magnetic Resonance Microscopy of Flows and Compressions of the Circulatory, Respiratory, and Digestive Systems in Pupae of the Tobacco Hornworm, *Manduca sexta*

**DOI:** 10.1673/031.008.1001

**Published:** 2008-02-18

**Authors:** Kevin J. Hallock

**Affiliations:** Center for Biomedical Imaging, Boston University School of Medicine, 650 Albany Street, X-B05B, Boston, MA, 02118

**Keywords:** hemolymph flow, ceolopulses

## Abstract

Circulatory, respiratory, and digestive motions in *Manduca sexta* pupae were observed using proton-density weighted and fast-imaging with steady-state free procession magnetic resonance microscopy. Proton-density weighted images clearly differentiated pupal air sacs from the hemolymph and organs because, as expected, the air sacs appeared dark in these images. Steady-state free procession imaging allowed real-time monitoring of respiration and circulation, creating movies of hemolymph circulation. Some of the movies show compression and inflation of the air sacs as well as abdominal movements consistent with previously reported ceolopulses. To our knowledge, this is the first magnetic resonance microscopy study of insect circulation and respiration and these preliminary results demonstrate the potential of magnetic resonance microscopy for studying *in vivo* dynamic processes in insects.

## Introduction

As a noninvasive imaging method that can produce high resolution images of internal anatomy, magnetic resonance imaging, known as MRI, revolutionized medicine. Magnetic resonance microscopy (MRM) is similar to magnetic resonance imaging, but is focused on studying smaller samples and anatomy; however, it is not as widely known as magnetic resonance imaging. To date, MRM has been used to examine a wide variety of samples, including plants (Narasimhan et al. 2005), small mammals (Narasimhan et al. 2005), and insects (Skibbe et al. 1995; Jasanoff et al. 2002; Wecker et al. 2002; Favila et al. 2004). Since MRM is a nondestructive and noninvasive imaging technique, the same specimen can be repeatedly imaged, unlike classical histological methods that require the destruction of the specimen.

A recent review of MRM in entomology provided an excellent overview and discussion of MRM's applications and limitations with regard to insects (Hart et al. 2003). One of the major limitations of structural MRM as discussed by Hart et al. is motion; internal motions during an MRM experiment can reduce the quality of the image, often resulting in blurring and loss of detail when the experimental time is long compared to the timescale of the motion (e.g. see Figure 2 in Hart et al. 2003). While motion limits the quality of anatomical MRM images, the fact that MRM is influenced by motion can be an advantage when looking at dynamic processes such circulation and respiration.

The goal of this work was to assess the potential of MRM in studying insect circulation and respiration. Investigations of these systems would provide a better understanding of this diverse class of animals, and as this report shows, MRM can make important contributions to this end.

## Materials and Methods

### Animals

Seven *Manduca sexta* L. (Lepidoptera: Sphingidae) pupae were purchased from Carolina Biological Supply (Burlington, NC, www.carolina.com) and stored in the dark at room temperature for the duration of these studies. Each pupa was 5–6 cm is length and 1–1.5 cm in diameter and each one was stored in a separate container. The pupae were only removed from their containers for the MRM experiments, and then were returned after the experiments were completed. Two of the imaged pupae died for unknown reasons.

### Magnetic Resonance Microscopy

**Figure 1.**
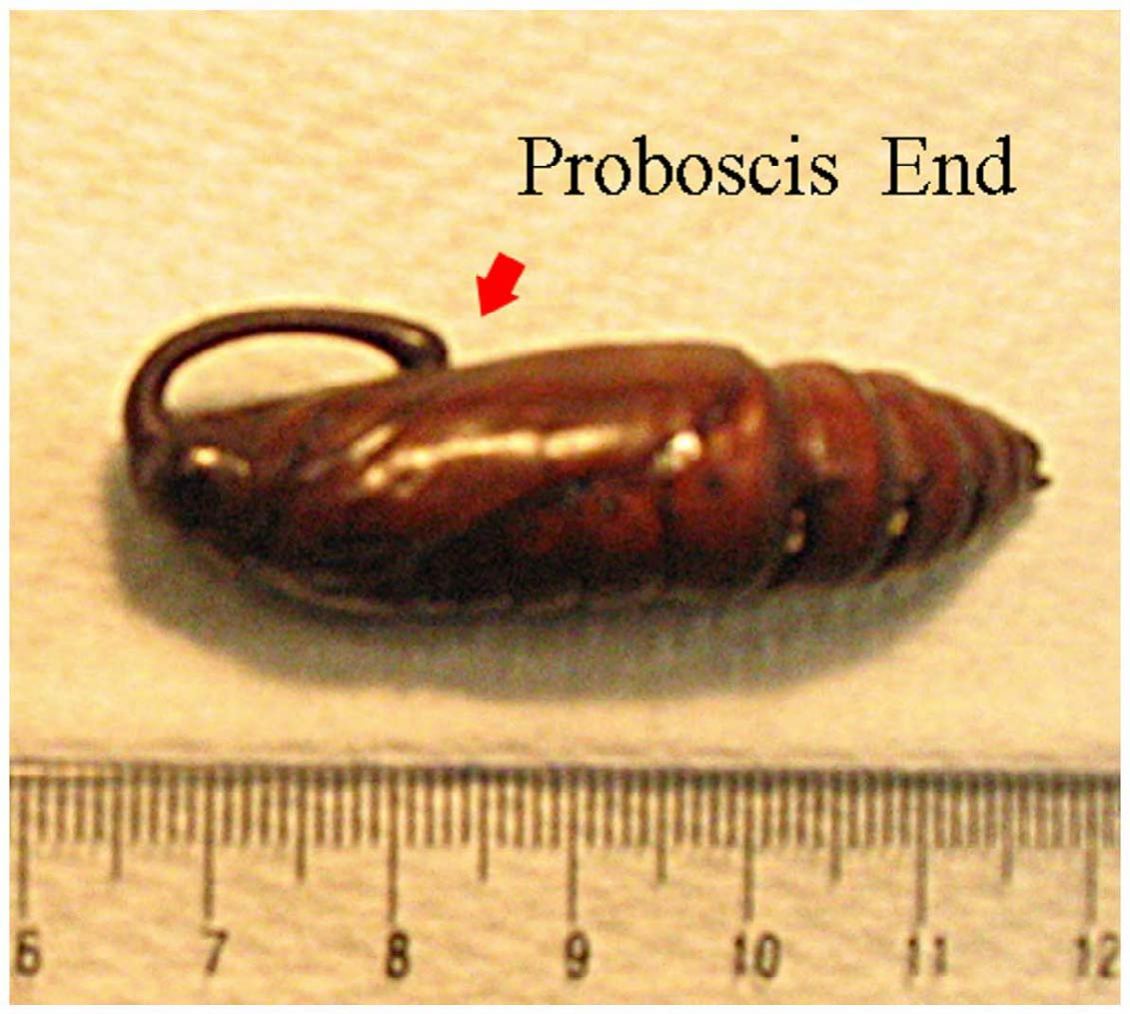
Pupa of *Manduca sexta*. The red arrow highlights where the proboscis joins the exoskeleton, which is used as an anatomical reference point for all of the images because it is easily located using MRM.

MRM experiments were performed using a Bruker 500 MHz instrument operating at a magnetic field of 11.7 T with a resonance frequency of 500.15 MHz (1H). Images were obtained using a commercial Bruker probe fitted with a 20 mm birdcage coil. Proton-density weighted (PDW) images were acquired using a standard spin echo experiment (Angle of excitation pulse (α) =90°, Echo Time (TE) = 15 ms, Repetition Time (TR) = 1s, Number of averages (NEX) = 1). Each PDW experiment required about 2 minutes to acquire. Dynamic imaging of the internal motions was accomplished using a balanced FISP sequence (α = 60°, TE = 1.239 ms, TR =2.468 ms, NEX = 1). Each fast-imaging with steady-state free procession (FISP) acquisition was preceded by 5000 dummy scans to ensure a steady-state was created before data acquisition began. Sixty consecutive image frames were then acquired, with one frame requiring 316 ms to obtain. The frames were then combined into a movie. All images had a field of view = 20 mm × 20 mm, matrix = 128 × 128 (yielding a 156 µm in-plane resolution), and slice thickness = 500 µm. During preliminary work, multiple FISP experiments were continuously run; however, the pupa wriggled out of the field of view after several minutes of FISP imaging. The pupa's movement was probably caused by experimental noise as this FISP experiment was louder than other MRM sequences, but other possibilities cannot be ruled out, such as heating of the pupa. To avoid this complication, a 15 minute gap was placed between each FISP experiment reported in this work. Future work may allow for this gap to be reduced. Each slice's location will be stated relative to the most posterior location of the proboscis, where the proboscis joins the exoskeleton (the red arrow in Figure 1). This location was chosen because it is easily identified in the MRM images of each pupa.

Throughout each MRM experiment, a pupa was placed vertically in the coil, buried in sand (J.T. Baker, Philipsburg, NJ, www.jtbaker.com) to reduce motion, and maintained at 25°C; enough sand was used to cover the pupa by 1–2 cm. In-between every FISP experiment, a PDW image was obtained to check for pupal movement. With this experimental design, whole-body motions were rare and always small (less than 1mm). When whole-body motions did occur, the most recent PDW image was used for comparison, although the pupae shown in this work did not move during the experiments. Because little motion occurred when these pupae were buried in sand, other investigators may wish to consider using sand as a restraint for MRM of other species. Before and after each experiment, the imaged pupa was visually inspected to make certain it was alive.

### Brief Overview of MRM

A brief overview of MRM focusing on the basics required to understand this paper is presented. For a more detailed explanation of MRM, the reader is encouraged to read Hart et al. (2003), and for more MRM theory, the book by Callaghan (1991). MRM is similar to magnetic resonance imaging (MRI) and nuclear magnetic resonance spectroscopy (NMR). MRM requires that a sample be placed in an external magnetic field. Protons (1H) are then excited using a radiofrequency pulse. Protons are the most common nuclear isotope used in magnetic resonance and were the only one used in this report. Although many molecules have protons, water and fat are the most likely sources of 1H magnetic resonance signals because of their high concentrations in living organisms, compared to proteins, DNA, metabolites, etc. Many rigid structures, such as chitin, also have protons, but these require specialized imaging methods to be observed and appear dark in the images presented in this paper.

Two techniques were used to examine insects in this report: proton-density weighted (PDW) imaging and fast-imaging with steady-state free procession (FISP). The intensity in PDW images is related to the concentration of protons, so an area containing more protons has higher signal intensity and is brighter in the image. Because an insect's air sacs contain relatively few protons, they will appear dark in a PDW image, assuming the air sac undergoes no motion during the imaging experiment. FISP is a more complex technique, but as with other steady-state techniques, nuclei that flow into the field of view are usually brighter than nuclei that stay in the field of view. Quantitating flow using FISP is difficult (Markl et al. 2003; Markl et al. 2004), but qualitatively, bright areas indicate fluid flowing into the field of view. All of the movies presented in this report were made using FISP. For this report, the field of view is a single imaging slice obtained from an *M. sexta* pupa and the hemolymph or gut contents are the most likely sources of flowing protons. The location of each image's field of view is described on Table 1.

**Table 1.**
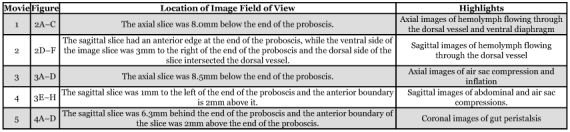
Summary of movies and images. Video clips can be accessed at http://digital.library.wisc.edu/1793/23222

**Figure 2.**
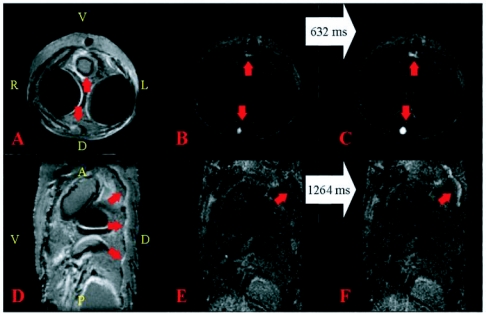
Figure 2. Images of a 20-day old *Manduca sexta* pupa. The orientation of the PDW and their corresponding FISP images is identical, so the orientation is only displayed on the former. For the PDW images: A = Anterior, D = Dorsal, L = Left, P = Posterior, R = Right, V = Ventral. The white arrows connecting the FISP images indicate the time that elapsed between the displayed images. A) Axial PDW image. The red arrow is pointing towards the location of the dorsal vessel. The two large dark areas on the right and left side of the body are the air sacs. The slice location of A—C was 8.0 mm below the end of the proboscis. B—C) Individual frames from Movie 1; the frames were acquired 632 ms apart. The red arrows in both images point towards the dorsal vessel and ventral diaphragm, respectively. D) Sagittal PDW image. The dark area ventral to the middle arrow is part of an air sac. The slice location of D had an anterior edge that began at the end of the proboscis. The ventral side of the slice is 3 mm to its right, while the dorsal side of the slice intersected the dorsal vessel. The red arrows are pointing towards the location of the dorsal vessel. E—F) Individual frames from Movie 2 at the same location and orientation as (D); the frames were acquired 1264ms apart. The red arrow in both images point towards where hemolymph is observed flowing through the dorsal vessel in (F). See also Movie 1 and Movie 2. **Movie 1**. Each frame is separated by 316 ms. Movie 1 corresponds to Figure 2A-C. See Figure éfor orientation and structural information. Hemolymph flow through the dorsal vessel and ventral diaphragm is seen throughout the movie. This video clip can be accessed at http://digital.library.wisc.edu/1793/23227 **Movie 2**. Each frame is separated by 316 ms. Movie 2 corresponds to Figure 2D—F. See Figure 1 for orientation and structural information and the text for a detailed description. Several pulses of hemolymph flowing through the dorsal vessel are observed on the right side of the movie, which is the dorsal side of the pupa. This video clip can be accessed at http://digital.library.wisc.edu/1793/23226

## Results and Discussion

### Monitoring Motion Using MRM

Figure 2 shows representative MRM images obtained from an *M. sexta* pupa 20 days after pupation. Similar images were obtained from pupae of different ages, and show the air sacs fully inflated with hemolymph flowing through the dorsal vessel and ventral diaphragm. Figure 2A shows the dorsal vessel (dorsally-pointing red arrow) and the gut (ventrally-pointing red arrow). The dark areas on the right- and left-hand sides are the inflated air sacs. Figures 2B and C show individual frames from Movie 1 that were collected 632 ms apart. In both images, the ventrally-pointing red arrow aims at hemolymph flow, most likely through the ventral diaphragm, while the dorsally-pointing arrow aims at the rhythmic flow through the dorsal vessel. Movie 1 shows that the pulsating dorsal vessel brought fresh hemolymph into the field of view, while the ventral diaphragm exhibited less flow, but was still active. Additional areas of brightness sometimes appeared in the movie as well, including some thin bright areas on the inner sides of the air sacs, but this flow was small and limited in both duration and volume.

**Figure 3.**
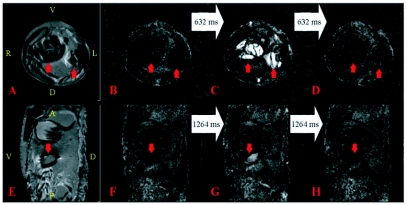
Images of a 13-day old *Manduca sexta*, pupa. The orientation of the PDW and their corresponding FISP images is identical, so the orientation is only displayed on the former. For the PDW images: A = Anterior, D = Dorsal, L = Left, P = Posterior, R = Right, V = Ventral. The white arrows connecting the FISP images indicate the time that elapsed between the displayed images. A) Axial PDW image. The red arrows point towards the location of the air sacs. The slice location of A—D was 8.5 mm below the end of the proboscis. B—D) Individual frames from Movie 3 at the same location and orientation as (A); the frames were acquired 632 ms apart. The red arrows in the images identify the location of the air sacs. The intensity in (C) suggests the air sacs compressed. E) Sagittal PDW image. The red arrow identifies part of an air sac. The slice location of E—H was 1mm to the left of the end of the proboscis and the anterior boundary is 2mm above it. F—H) Individual frames from Movie 4 at the same location and orientation as (E); the frames were acquired 1264 ms apart. The red arrow in the images identifies the air sac and the hemolymph that occupies the same volume temporarily (G). See also Movie 3 and Movie 4. **Movie 3**. Each frame is separated by 316 ms. Movie 3 corresponds to Figure 3A-D. See Figure 3A for orientation and structural information and the text for a detailed description. Two cycles of compression and inflation of the air sacs are seen about halfway through the movie. This video clip can be accessed at http://digital.library.wisc.edu/1793/23225 **Movie 4**. Each frame is separated by 316 ms. Movie 4 corresponds to Figure 3E—H. See Figure 3E for orientation and structural information and the text for a detailed description. Several cycles of compression and inflation of the air sacs as well as abdominal motion are seen during the first half of the movie. This video clip can be accessed at http://digital.library.wisc.edu/1793/23224

Figures 2D—F shows images obtained from a sagittal image slice. The dorsal vessel is highlighted by the three red arrows in Figure 2D. In this sagittal PDW image, the dorsal vessel appears as a squiggly area of slightly different intensity than the rest of the pupa. Figures 2E—F are frames from Movie 2 that show hemolymph flowing through the dorsal vessel. In Figure 2E, the dorsal vessel is dark, while Figure 2F has a squiggly bright area. Movie 2 demonstrates the flow of hemolymph through the dorsal vessel much better than the individual frames shown in Figure 2. These movies nicely demonstrate the inflated air sacs, which remain almost immobile, and the pulsatile flow of hemolymph through the dorsal vessel.

**Figure 4.**
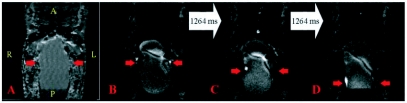
Images of a 21-day old *Manduca sexta* pupa. The orientation of the PDW and their corresponding FISP images is identical, so the orientation is only displayed on the former. For the PDW images: A = Anterior, D = Dorsal, L = Left, P = Posterior, R = Right, V = Ventral. The white arrows connecting the FISP images indicate the time that elapsed between the displayed images. A) Coronal PDW image. The red arrows identify part the gut. The slice location of B—D was 6.3 mm behind the end of the proboscis and the anterior boundary is 2 mm above it. B—D) Individual frames from Movie 5 at the same location and orientation as (A); the frames were acquired 1264 ms apart. The red arrows in the images identify gut and follow a peristaltic wave moving in the posterior direction. See also Movie 5. **Movie 5**. Each frame is separated by 316 ms. Movie 5 corresponds to Figure 4A—D. See Figure 4A for orientation and structural information and the text for a detailed description. Peristalsis of the gut is visible in the opening frames of the movie. This video clip can be accessed at http://digital.library.wisc.edu/1793/23223

In addition, Figure 2 and Movies 1–2 demonstrate that the brightest signals in these FISP experiments were caused by flowing fluids. In this case, hemolymph was the source of the bright signal, but later images show that peristalsis of the gut can also appear bright. Areas such as the air sacs that contain no observable protons remained dark during a FISP experiment, suggesting that when signal was observed in the air sac region (Figure 3), hemolymph had flowed into the area vacated by the compressing air sac.

Figure 3A shows an axial PDW image with the red arrows pointing towards the air sacs. Unlike the air sacs shown in Figure 2A, these are not dark, but they are less intense than the surrounding tissue. From an experimental perspective, this could be caused by an air sac that was partially-compressed for the entire duration of the two minute experiment, or by the rapid compression and inflation of the air sacs during the experiment. The latter would yield an average of the empty and full air sac signals. Unlike FISP, the time resolution of this PDW method did not allow for differentiating the two possibilities. Figures 3B—D show a sequence of frames from Movie 3, each 632 ms apart. Initially, the air sacs were filled with air (Fig. 3B), a bright signal then appeared in the air sac region indicative of flow (Figure 3C), and then the air sacs became dark again (Fig. 3D). About halfway through Movie 3, two sets of bright signals were observed in rapid succession (1264 ms apart); the rest of the time the air sacs remained dark similar to Figure 2A and Movie 1. Hemolymph also flowed through the dorsal vessel during the pulsations.

Figure 3E is a sagittal image similar to Figure 2G, except it has some intensity in the middle of the air sac's dark area (red arrow). As described previously for Figure 3A, this intensity could have resulted from a partially-compressed air sac that was static during the entire imaging experiment, or from air sac motion during the two minute imaging experiment. Figures 3F—H are frames from Movie 4, each obtained 1264 ms apart. These images show that the air sac was initially empty (Figure 3F), then fluid entered the air sac area (Fig. 3G), and then the air sac area was once again empty 1264 ms later (Figure 3H). In addition to showing this flow, Movie 4 reveals additional abdominal movement in the lower part of the image. This movement did not involve the entire body because the anterior side remained static throughout the movie; this is especially noticeable on the ventral side of the image. While the anterior side remained unperturbed, the organs posterior to the air sac also compressed. These motions are consistent with the previously described ceolopulses (Sláma 2000), although additional work is required to better characterize the cause the observed motions.

Figure 4A is a coronal PDW image and the red arrows point to the pupa's gut. The FISP images (Figure 4B—D) are frames from Movie 5 that were each 1264 ms apart. In Figure 4B—D, the red arrows follow a peristaltic pulsation as it moved from the upper to the lower gut, but this is more clearly seen in the opening frames of Movie 5.

The movies and images presented in this paper are summarized in Table 1. These results demonstrate that MRM can be used to characterize the internal motions of insects. MRM easily differentiates between fluid-filled cavities and an insect's air sacs (Figure 2), making it possible to monitor changes in air sac volume in real time, as well as the circulation of hemolymph through the dorsal vessel and the ventral diaphragm. Of these, the observed flow into the air sac region (Figure 3C & Movie 3) is the largest movement of hemolymph reported in this paper. Movie 3 is consistent with *M. sexta's* air sacs undergoing two complete compression and expansion cycles over the course of a few seconds and then returning to a static position. This motion may indicate a rapid convective gas exchange (Sláma 1999; Harrison 2003). In Movie 4, motions restricted to the abdomen were observed, consistent with previously reported ceolopulses (Sláma 2000). In addition to circulation and respiration, peristalsis of the gut was observed in Movie 5 (and Figure 4A—D). To our knowledge, this is the first MRM study to investigate insect circulation and respiration and even though preliminary, the results are encouraging.

In conclusion, MRM is a promising tool for investigating internal motions of insects. And because it is noninvasive, it can be combined with other methods and used to follow the same insect through its life cycle. Additional MRM development is needed to extend the methods presented here to include quantitative analysis, insect-specific MRM sequences, as well as insect-specific MRM hardware. Magnetic resonance imaging has revolutionized how dynamical studies are conducted in humans and MRM can do the same for insect research.

## Abbreviations

FISP -: fast-imaging with steady-state free procession
MRM -: magnetic resonance microscopy
NEX -: number of averages
PDW -: proton-density weighted
TE -: echo time
TR -: repetition time
